# Multimodal neuroimaging of alcohol use: from acute neurochemical effects to chronic brain network reorganization and precision treatment targets

**DOI:** 10.3389/fneur.2026.1798789

**Published:** 2026-05-11

**Authors:** Rodolfo Ferrando

**Affiliations:** 1 Nuclear Medicine and Molecular Imaging Center, Hospital de Clínicas, Universidad de la República, Montevideo, Uruguay; 2 Centro Uruguayo de Imagenologia Molecular (CUDIM), Montevideo, Uruguay

**Keywords:** adolescence, alcohol use disorder (AUD), magnetic resonance imaging, molecular imaging, neuroimaging (anatomic and functional), positron emission tomograghy (PET), precision medicine, vulnerability

## Abstract

Alcohol use and alcohol use disorder (AUD) remain among the leading causes of preventable morbidity and mortality worldwide, yet the mechanisms by which alcohol alters brain structure and function across the lifespan are only partially understood. Over recent decades, multimodal neuroimaging has provided key insights into the acute neurochemical and hemodynamic effects of alcohol, the neuroadaptations associated with sustained exposure, and the circuits that mediate vulnerability, progression, and relapse. In this narrative review, we integrate evidence from structural and diffusion MRI, task-based and resting-state fMRI, magnetic resonance spectroscopy, perfusion and metabolic techniques (arterial spin labeling, perfusion SPECT, FDG PET, and ^15^O PET), and molecular PET/SPECT targeting dopaminergic and non-dopaminergic systems. Acute alcohol effects include regionally specific changes in cerebral blood flow and metabolism, perturbations in sensory processing and cognitive control networks, and rapid modulation of dopaminergic, GABAergic, glutamatergic, and opioid signaling, with transient neuroimmune changes. Chronic exposure is associated with macro- and microstructural injury, large-scale network reorganization, and persistent molecular abnormalities in reward, salience, and executive control circuits, with links to craving severity and relapse risk, including heterogeneous neuroimmune findings and reduced synaptic integrity. We also highlight adolescent alcohol use, where exposure during ongoing neurodevelopment is associated with deviations in cortical and white-matter maturation and altered task-related brain responses, with potential lasting effects on connectivity and cognition. Finally, we summarize characteristic imaging patterns of alcohol-related neurological syndromes, highlight sex-related differences, review imaging markers of vulnerability and prognosis (familial risk, genetic variation, and predictors of treatment response), and outline priorities for precision medicine in AUD, emphasizing longitudinal multimodal designs, harmonization, and the development of validated molecular targets and imaging-guided interventions.

## 1 Introduction

Over recent decades, neuroimaging has become central to neuroscience, allowing *in vivo* study of brain structure and function with high spatial resolution. Together with developments in neuroanatomy, neurophysiology and data science, these techniques have made it possible to relate specific cortical regions and large-scale networks to well-defined cognitive and behavioral domains. Neuroimaging can detect functional and even molecular alterations in the same regions where histopathological changes were described more than a century ago, often long before overt anatomical damage or clinical symptoms become apparent (1, 2). Progress in neuropharmacology and radiopharmacy has enabled the analysis of cerebral metabolic processes and the pharmacodynamics of key neurotransmitter systems—including dopamine, GABA, serotonin, glutamate and opioids—as well as the *in vivo* visualization of other molecular targets such as abnormal protein deposits, neuroinflammation and synaptic density (1–3). As a result, neuroimaging is now both a powerful tool for basic research and a key resource for designing diagnostic strategies and developing new treatments.

Alcohol consumption is one of the main contributors to the global burden of disease and premature mortality, and alcohol use disorder (AUD) is among the most prevalent and disabling psychiatric conditions worldwide (4, 5). Alcohol produces neurotoxic and neuroadaptive effects that impact multiple brain systems. Structural and functional MRI, together with PET and SPECT, make it possible to investigate directly in the human brain both the acute effects of alcohol ingestion and the cumulative structural and functional changes associated with chronic use and dependence (1–3). These multimodal studies have improved our understanding of the mechanisms underlying the reinforcing effects of alcohol, the neuroadaptations associated with prolonged exposure, and the alterations in frontostriatal, frontocerebellar and limbic circuits that sustain heightened motivation to drink and loss of control, which are hallmarks of AUD (1–3). Longitudinal imaging has also been used to assess structural and functional recovery during abstinence and to explore the prognostic value of neuroimaging measures for clinical outcomes (2, 6).

Neuroimaging is equally important for characterizing the clinical spectrum of alcohol-related brain damage. Structural MRI is essential for detecting and diagnosing neurological syndromes associated with prolonged alcohol use, such as Wernicke’s encephalopathy, Korsakoff’s syndrome and other forms of alcohol-related brain damage, which show distinctive patterns of regional atrophy and signal change (1, 7). At the same time, functional and molecular imaging have begun to elucidate the neuronal mechanisms by which genetic and environmental factors confer vulnerability to heavy drinking and AUD, including alterations in dopaminergic, opioid and other neuromodulatory systems that influence reward sensitivity, stress responsivity and cognitive control (1–3, 8, 9).

Importantly, alcohol exposure occurs within a dynamic brain. In adolescence and emerging adulthood, when cortical thinning, white matter maturation and network refinement are still ongoing, heavy or binge drinking has been associated with deviations in structural development and connectivity, and with alterations in functional responses during cognitive and emotional tasks (10, 11). These changes may contribute to increased vulnerability to subsequent substance use disorders and other psychiatric outcomes. At the other end of the lifespan, chronic heavy drinking interacts with aging and comorbid medical conditions, exacerbating brain atrophy and network disruption and contributing to alcohol-related cognitive impairment and dementia (1, 2, 6, 7, 12).

Beyond describing average differences between groups of drinkers and non-drinkers, recent work has focused increasingly on identifying neuroimaging markers of vulnerability. Studies in individuals at familial or genetic risk, as well as in detoxified patients, have linked individual differences in frontostriatal and limbic structure and function to risk of developing AUD, severity of craving, probability of relapse and response to pharmacological or psychosocial treatments (8, 11, 13–15). Together, these converging lines of evidence highlight the potential of multimodal neuroimaging to support a more mechanistic, circuit-based understanding of AUD.

The current DSM-5 framework conceptualizes AUD as a single condition distributed along a severity continuum, rather than as separate categories such as abuse and dependence. This dimensional perspective is particularly relevant for neuroimaging studies, which often capture variation in alcohol exposure, symptom burden, and clinical course that does not fit neatly into categorical distinctions. In this narrative review, we synthesize and integrate findings from structural, functional, perfusion, spectroscopic and molecular neuroimaging across the spectrum of alcohol exposure and clinical course—from acute intoxication and binge/heavy drinking to established AUD, abstinence, and relapse—with additional attention to adolescent use, sex-related differences, alcohol-related neurological syndromes and biomarkers of vulnerability and prognosis. We also highlight methodological challenges and future directions toward the use of neuroimaging as a platform for precision treatment strategies in AUD.

This narrative review was based on a targeted literature search of PubMed/MEDLINE and Scopus supplemented by manual review of reference lists of relevant articles, focusing on structural, functional and molecular neuroimaging studies in alcohol use and AUD. Search terms included combinations of “alcohol,” “alcohol use disorder”, “binge drinking,” “abstinence,” “withdrawal,” “acute,” “chronic,” “neuroimaging,” “MRI,” “DTI,” “MRS”, “ASL”, “fMRI”, “connectivity”, “FDG PET”, “receptor PET” and “SPECT”, as well as molecular targets relevant to addiction (e.g., dopamine, opioid, GABA, glutamate, TSPO, SV2A). Priority was given to human studies, meta-analyses and well-characterized clinical or translational investigations, while seminal and illustrative reports were included when relevant to support specific mechanistic concepts. Given the narrative scope of the review, no formal systematic review methodology was applied. The selection aimed to provide an integrative and clinically oriented overview of both established findings and emerging approaches across imaging modalities.

## 2 Neuroimaging modalities in alcohol research

Neuroimaging studies of alcohol use build on a set of complementary techniques that capture different aspects of brain structure, perfusion, function and chemistry. Below we briefly summarize the main modalities that will be referred to in subsequent sections, with an emphasis on the type of information they provide in the context of alcohol research.

### 2.1 Structural imaging

Computed tomography (CT) provided some of the earliest *in vivo* descriptions of alcohol-related brain damage, including diffuse cortical atrophy and ventricular enlargement, with partial reversibility after abstinence reported in early studies. In current practice, its role in alcohol-related conditions is mainly structural screening and acute care, allowing rapid exclusion of hemorrhage, trauma, or other macroscopic complications, while also identifying atrophy patterns associated with prolonged alcohol exposure.

Structural MRI allows high-resolution *in vivo* characterization of brain anatomy and is the main technique for assessing alcohol-related structural brain changes. In this context, it enables detailed evaluation of regional atrophy patterns, volumetric and morphometric abnormalities, and the diagnosis of specific alcohol-related neurological syndromes. Its high tissue contrast and quantitative capabilities also make it the preferred modality for tracking structural change over time, including partial recovery with abstinence. Morphometric MRI methods provide quantitative assessment of interindividual anatomical differences. Voxel-based morphometry (VBM) estimates regional gray and white matter differences on a voxel-wise basis, whereas deformation-based morphometry (DBM) quantifies local shape changes. These approaches are useful for detecting subtle abnormalities and relating them to clinical or cognitive variables.

Diffusion tensor imaging (DTI) is an MRI modality sensitive to white matter microstructure and is particularly useful for characterizing alcohol-related changes in structural connectivity. The most commonly reported metrics are fractional anisotropy and mean diffusivity, which provide complementary information about fiber organization and tissue integrity. DTI can detect white matter abnormalities even when conventional MRI appears normal.

### 2.2 Functional MRI and perfusion MRI

Functional MRI (fMRI) detects blood-oxygenation level–dependent (BOLD) signal changes as an indirect marker of neural activity. In alcohol research, task-based fMRI has been widely used to study cue reactivity, craving, inhibitory control, and decision-making, whereas resting-state fMRI characterizes intrinsic connectivity within and between reward, salience, and prefrontal control networks (1–3). Perfusion MRI is typically performed with arterial spin labeling (ASL), which provides a quantitative, non-invasive measure of regional cerebral blood flow. In alcohol research, ASL has been used to capture both acute alcohol-induced changes in perfusion and more chronic patterns of hypoperfusion in vulnerable networks.

### 2.3 Magnetic resonance spectroscopy

Proton magnetic resonance spectroscopy (MRS) provides non-invasive *in vivo* measures of brain metabolites related to neuronal integrity, membrane turnover, glial state, energy metabolism, and excitatory–inhibitory balance. In alcohol research, the most commonly reported metabolites include N-acetylaspartate (NAA), choline-containing compounds (Cho), total creatine (tCr), myo-inositol (mI), gamma-aminobutyric acid (GABA), and glutamate/glutamine (Glx) (1–3).

### 2.4 SPECT and PET

Single-photon emission computed tomography (SPECT) provides tomographic measures of tracer distribution in the brain. In alcohol research, perfusion SPECT with tracers such as 99mTc (99mTc-ECD and 99mTc-HMPAO) has been used to assess regional cerebral blood flow, whereas other ligands allow evaluation of dopaminergic markers such as the dopamine transporter and D2/3 receptors.

Positron emission tomography (PET) provides tomographic measures of regional metabolism, perfusion, and molecular targets. In alcohol research, ^18^F-FDG PET has been widely used to assess regional glucose metabolism, ^15^O-H_2_O PET to quantify cerebral blood flow, and receptor or transporter radioligands to probe neurotransmitter systems. More recently, PET tracers targeting the 18-kDa translocator protein (TSPO), a marker related to glial/neuroimmune responses, and synaptic vesicle glycoprotein 2A (SV2A), a marker of synaptic density, have extended this approach to neuroimmune and synaptic aspects of alcohol-related brain changes. Together, these PET approaches have been instrumental in characterizing acute and chronic alterations within reward, salience, and prefrontal control circuits (2, 3).

Across these modalities, studies can be broadly divided into baseline or observational designs and challenge paradigms, the latter examining dynamic brain responses to interventions such as alcohol, amphetamine, benzodiazepines, or alcohol-related cues. This distinction is particularly relevant when interpreting molecular imaging findings in AUD.

## 3 Acute brain effects of alcohol exposure

Acute alcohol intoxication produces rapid and regionally specific changes in cerebral perfusion, metabolism, neural activity and neurochemistry. These effects reflect the combined action of alcohol on vascular tone, synaptic transmission and network dynamics, and can be captured with perfusion and metabolic imaging, task-based fMRI, magnetic resonance spectroscopy and molecular PET (1, 16). Acute (and early-withdrawal) neuroimaging findings across modalities are summarized in Table 1.

**Table 1:** Acute effects of alcohol use on neuroimaging.

Modality	Specific technique (metric)	Key regions/networks	Window/condition	Comment	References
Perfusion/metabolism	ASL (rCBF)	mOFC/OFC ↑ (dose-dependent); cerebellum/posterior regions ↓ at higher doses	Mild–moderate intoxication (minutes–hours)	Rapid vasomotor and reward/salience response; usually reversible	(17)
	SPECT (^133^Xe) (rCBF)	Frontal rCBF changes (↑/↓; dose- and time-dependent)	Intoxication (minutes–hours)	State-sensitive; typically rapidly reversible	(16, 20)
	^18^F-FDG PET (CMRglc)	Global CMRglc ↓; greatest relative reductions in cerebellum and occipital cortex	Intoxication (hours)	Overall glucose metabolism decreases during intoxication; recovery over days	(18, 19)
	^11^C-acetate PET (Acetate uptake)	Cerebellum ↑ (prominent)	Intoxication (hours)	Shift toward acetate as an energy substrate during intoxication	(18)
Functional MRI	Task-based fMRI (BOLD)	Sensory processing: sensory cortex ↓; executive control: dlPFC/dACC ↓; threat/negative emotion processing: amygdala/vmPFC ↓	During task performance under intoxication (minutes–hours)	State-dependent network effects; task- and dose-specific	(20–30)
MRS	^1^H-MRS (GABA, Glu/Glx, NAA, Cho)	Occipital: GABA ↓ and NAA ↓ (IV ethanol); frontal: Cho ↑.	Intoxication (hours)	Inhibitory/metabolic changes in intoxication; excitatory rebound in withdrawal	(31–33)
Molecular imaging (PET)	Dopamine (^11^C-raclopride) (Dopamine release (↓BP_ND_))	Ventral striatum/NAc: dopamine ↑ (binding ↓)	IV infusion/priming/intake (minutes–hours)	Mesolimbic reinforcement signal; rapid and reversible	(34–36)
	μ-opioid (^11^C-carfentanil) (Change in BP_ND_)	OFC and ventral striatum/NAc: opioid release ↑ (binding ↓)	Intake (minutes–hours)	Hedonic/opioid reinforcement; reversible within hours	(37)
	TSPO (^18^F-DPA-714) (Binding (V_T_))	Widespread cortical/subcortical ↑	Acute heavy exposure (hours–days; animal model)	Early neuroimmune response; partial reversibility possible	(38, 39)

ACC, anterior cingulate cortex; ASL, arterial spin labeling; AUD, alcohol use disorder; BP_ND_, binding potential relative to non-displaceable uptake; CBF, cerebral blood flow; CMRglc, cerebral metabolic rate of glucose; dACC, dorsal anterior cingulate cortex; dlPFC, dorsolateral prefrontal cortex; FDG, fluorodeoxyglucose; fMRI, functional MRI; Glu, glutamate; Glx, glutamate + glutamine; IV, intravenous; MRS, magnetic resonance spectroscopy; NAA, N-acetylaspartate; NAc, nucleus accumbens; mOFC, medial orbitofrontal cortex; OFC, orbitofrontal cortex; PET, positron emission tomography; rCBF, regional cerebral blood flow; SPECT, single-photon emission computed tomography; TSPO, 18-kDa translocator protein; vmPFC, ventromedial prefrontal cortex; V_T_, total distribution volume.

### 3.1 Cerebral blood flow and metabolism

Under acute intoxication, perfusion and metabolic studies converge in showing that alcohol intake rapidly alters brain physiology. ASL studies indicate that moderate alcohol intake increases cerebral blood flow (CBF) in several frontal regions, with particularly robust changes in medial prefrontal cortex and orbitofrontal cortex (OFC). However, these CBF increases are attenuated in individuals with a low subjective response to alcohol—that is, those who require higher doses to feel intoxicated—a phenotype associated with elevated risk for developing AUD (17). In contrast, FDG-PET consistently shows global decreases in brain glucose metabolism during intoxication, with the largest relative reductions in cerebellum and occipital cortex (18, 19). In parallel, PET studies have described increased cerebral acetate uptake, most prominently in cerebellum, suggesting an acute shift toward acetate as an alternative energy substrate when alcohol is present (18).

Overall, the evidence indicates that CBF and metabolism do not always change in the same direction or follow the same regional distribution. In several studies, alcohol has produced CBF increases in frontal and limbic cortices alongside metabolic decreases in occipital cortex and cerebellum (16, 19, 20). Beyond the acute shift in energy substrate toward acetate, part of this pattern reflects dose- and time-dependent vasodilatory and vasoconstrictive effects of alcohol on the cerebral vasculature (20). At moderate doses, CBF increases are predominantly frontal, whereas at higher doses reductions in cerebellar flow and, less consistently, in occipital cortex have been reported (17, 20).

From a neurochemical standpoint, frontolimbic CBF increases align with dopaminergic and opioid facilitation within reward and salience circuits, while decreases in cerebellum and posterior cortex are consistent with predominance of GABAergic over glutamatergic (NMDA) transmission, reduced metabolic demand and altered neurovascular coupling (2, 16). In line with this regionally divergent pattern, a moderate dose of alcohol (0.75 g/kg) produced a significant global decrease in glucose metabolism, with relative reductions in occipital cortex and cerebellum but relative increases in the striatum, including the nucleus accumbens (NAc), amygdala, insula and midbrain (20). This profile supports the notion that acute alcohol simultaneously suppresses posterior cortical processing and biases metabolic resources toward frontostriatal and limbic regions involved in reward, salience and motivational control.

### 3.2 Task-based fMRI

Task-based fMRI has been extensively used to characterize the impact of acute alcohol on neural responses during sensory, cognitive and affective processing. Early paradigms employing simple visual and auditory stimuli showed that alcohol dampens BOLD responses in primary sensory cortices, consistent with its general depressant effects on the central nervous system (20–22). In more complex cognitive tasks, such as working memory, mental rotation or response inhibition, acute intoxication systematically reduces activation in regions supporting executive control, including dorsolateral prefrontal cortex (dlPFC) and dorsal anterior cingulate cortex (ACC), and attenuates error-related signals and performance monitoring (23–26). These changes are often accompanied by increases in reaction time and greater variability in performance, even when accuracy is only mildly affected.

Driving simulator studies further illustrate that alcohol-related impairments in real-world behavior are paralleled by altered BOLD responses in frontal cortex, ACC, basal ganglia and cerebellum, with the degree of functional disruption correlating with decrements in driving performance (27). In affective paradigms, acute alcohol blunts amygdala and ventromedial prefrontal responses to negative or threatening stimuli and alters processing of emotional faces, effects that have been observed with both oral and intravenous administration (28–30). Overall, task-based fMRI indicates that even moderate intoxication compromises frontal, cingulate, and cerebellar circuits involved in attention, error monitoring and behavioral control, while dampening responsivity of limbic regions to negative emotional cues. Taken together, these findings show that BOLD signal changes are often more sensitive than observable performance measures, and that alcohol acutely disrupts control circuits even when behavioral output appears relatively preserved.

### 3.3 Magnetic resonance spectroscopy

Proton MRS provides a complementary view of the acute neurochemical effects of alcohol. Studies using intravenous ethanol infusion in healthy volunteers have reported rapid reductions in cortical GABA and NAA in occipital cortex, interpreted as potentiation of GABA_A_ receptor function accompanied by transient neuronal metabolic suppression (2, 31). In addition, acute alcohol exposure has been associated with transient increases in choline-containing compounds, suggestive of immediate alterations in membrane turnover and/or cell volume regulation (32, 33).

Although MRS lacks the molecular specificity of receptor PET, these findings are consistent with acute alcohol-induced neurochemical effects that combine transient cortical metabolic suppression with altered inhibitory tone. In this context, MRS provides a complementary metabolic perspective on intoxication that may help bridge cellular-level neurochemical changes with the functional alterations observed using other imaging modalities.

### 3.4 Molecular imaging

Molecular PET studies have provided direct evidence that acute alcohol increases dopamine levels in the human ventral striatum (34, 35). Using ^11^C-raclopride, a D2/3 receptor antagonist sensitive to competition with endogenous dopamine, robust decreases in striatal binding potential have been observed during intravenous ethanol infusion, corresponding to an average 10–15% increase in synaptic dopamine in the NAc (36). The magnitude of dopamine release correlates with the rapid onset of subjective effects and with ratings of stimulation and “high,” linking mesolimbic dopamine responses to the reinforcing properties of alcohol (16, 35, 36).

Beyond dopamine, acute alcohol intake also promotes the release of endogenous opioids. PET studies with *μ*-opioid receptor ligands (^11^C-carfentanil) have demonstrated alcohol-induced reductions in binding potential in OFC and ventral striatum, with larger effects in heavy or high-risk drinkers, suggesting that enhanced opioid responses may contribute to elevated reward valuation and risk for excessive use (37). These findings support the notion that acute co-activation of dopaminergic and opioid systems constitutes a key mechanism underlying the motivational value of alcohol and may promote patterns of excessive drinking.

More recently, experimental work using TSPO PET has begun to explore whether acute alcohol exposure elicits early neuroimmune responses. In a longitudinal study in adolescent baboons, a single episode of intense alcohol exposure triggered a widespread and sustained increase in ^18^F-DPA-714 binding throughout cortical and subcortical regions, with partial persistence at follow-up (38). Human data remain limited, but an acute oral alcohol challenge in social drinkers was likewise associated with a significant whole-brain increase in ^11^C-PBR28 signal (approximately 9–16%), with large effect sizes, together with concurrent alterations in peripheral cytokines, providing the first *in vivo* evidence in humans of a rapid brain immune response to alcohol (39). Together, these findings support the possibility that recent alcohol exposure may transiently increase TSPO PET signal, particularly during vulnerable developmental or biological states.

## 4 Chronic brain effects of alcohol exposure

### 4.1 Structural imaging

In chronic alcohol users, structural imaging has consistently documented volume loss affecting both gray and white matter, with particular vulnerability of frontal regions, ventricular enlargement and cerebellar involvement (1–3, 12, 40). A VBM meta-analysis confirmed gray-matter reductions in cortico–striato–limbic circuits, including dlPFC and precentral cortex, ACC, insula, superior temporal cortex, hippocampus, striatum and thalamus, compared with healthy controls (12). Other quantitative syntheses highlight decreases in white-matter volume and thinning of major tracts, especially the corpus callosum (2, 12, 41). A cross-modal overview of neuroimaging alterations in chronic alcohol use and AUD is provided in Table 2.

**Table 2:** Chronic effects of alcohol use on neuroimaging.

Modality	Specific technique (metric)	Key region/network (effect direction)	Abstinence timeframe	Neurobiological comment	References
Structural MRI	VBM/cortical thickness (Volume/thickness)	PFC, cerebellum, hippocampus ↓	Protracted (m–y)	Structural footprint; partly reversible	(1–3, 6, 12, 40–46)
	DTI (FA/MD) (FA/MD)	Frontal white matter & corpus callosum: FA ↓/MD ↑	Protracted (m–y)	Axonal disconnection; limited reversibility	(2, 47–50)
Functional MRI	Resting-state fMRI (Functional connectivity)	Control networks/DMN ↓; salience/reward ↑/reorganization	Early to protracted (d–m)	Network reconfiguration; partly reversible	(2, 55–58)
	BOLD (executive tasks) (BOLD reactivity)	dlPFC/ACC: hypoactivation or inefficiency	Early (d–w)	Control inefficiency; partly reversible	(2, 3, 15, 53, 54)
	BOLD (alcohol cue reactivity) (BOLD reactivity)	Striatum/ACC/medial PFC (incl. vmPFC) ↑	Early (d–w)	Cue-induced motivational salience; predicts craving/relapse; treatment-sensitive	(3, 13, 14, 51, 52)
Flow/metabolism	ASL (CBF)	PFC & cerebellum ↓	Early to protracted (w–m)	Sustained hypoperfusion; partly reversible	(59–62, 66, 67)
	FDG-PET (CMRglc)	Frontal/parietal/cingulate cortex ↓ (± thalamus/cerebellum)	Early (w)	Frontal hypometabolism; partly reversible	(3, 16, 59, 63–65)
MRS	^1^H-MRS (NAA/tCr, Cho/tCr, Glu/Glx (± mI/tCr))	Frontal/temporal/cerebellum/thalamus: NAA & Cho ↓; early abstinence: ACC Glu/Glx ↑ (tends to normalize)	Early to protracted (w–m)	Slow recovery (≈3–6 months); glutamate is dynamic	(1–3, 94–103)
Molecular imaging (PET)	D2/3 (^11^C-raclopride/^18^F-fallypride/^18^F-DMFP) (Availability (BP_ND_/V_T_))	Striatum ↓	Early to protracted (w–m–y)	Attenuated dopaminergic tone; partial reversibility	(16, 69, 73, 76)
	Dopamine synthesis (^18^F-DOPA) (Ki)	Striatum: no consistent group difference; lower ventral striatal uptake ↔ craving/relapse risk	Early (d–w)	Presynaptic synthesis capacity relates to clinical course	(71, 72)
	VMAT2 terminal markers (^11^C-DTBZ) (Binding (V_T_))	Striatum: terminal marker signal ↓ (limited evidence)	Early (w)	Reduced presynaptic terminal integrity; may contribute to blunted dopamine release	(74)
	μ-opioid receptor (^11^C-carfentanil) (Availability (BP_ND_))	Ventral striatum ↑ and/or frontal–parietal ↓ (heterogeneous); VS availability ↔ craving	Early abstinence (w)	Apparent increases may reflect reduced endogenous opioid tone; post-mortem supports μ-receptor loss	(16, 77, 78)
	CB1 (^18^F-FMPEP-d2, ^18^F-MK-9470) (Binding (V_T_/mSUV))	Widespread cortex/subcortex ↓	Early (w)	Reduced endocannabinoid tone; persists after ~4 weeks	(86, 87)
	mGluR5 (^11^C-ABP688) (Binding (DVR))	Amygdala & temporal lobe ↑ (≥25 days abstinent; non-smoking men)	Protracted (w–m)	Affective/motivational cue processing; higher availability ↔ lower temptation	(85)
	TSPO PET (^11^C-PBR28) (Binding (V_T_))	Global ↓ and/or hippocampus ↓ (detoxified AUD; heterogeneous)	Early (d–w)	No consistent increase; stage- and method-dependent TSPO signal	(88–92)
	SV2A PET (^11^C-UCB-J) (Availability (BP_ND_))	Frontal cortex, striatum, hippocampus ↓ (± cerebellum trend ↓)	Early to protracted (w–m)	Lower binding with greater severity; suggests reduced synaptic density	(93)
Molecular imaging (PET and SPECT)	SERT (binding ratio; cerebellar reference)	Brainstem/raphe projections ↓	Early (w)	Reduced serotonergic tone; linked to negative mood symptoms	(8, 84)
	GABA_A_/BZD (^123^I-iomazenil/^11^C-flumazenil) (BZD binding)	Frontal/ACC/cerebellum ↓ (6–20%; variable)	Early (w)	Cortical inhibitory dysregulation; abstinence-duration dependent	(9, 64, 79–83)

ACC, anterior cingulate cortex; ASL, arterial spin labeling; BOLD, blood oxygen level-dependent; BP_ND_, binding potential relative to non-displaceable uptake; BZD, benzodiazepine; CBF, cerebral blood flow; CMRglc, cerebral metabolic rate of glucose; CB1, cannabinoid type 1 receptor; Cho, choline-containing compounds; DAT, dopamine transporter; dlPFC, dorsolateral prefrontal cortex; DMFP, desmethoxyfallypride; DMN, default mode network; DTI, diffusion tensor imaging; DVR, distribution volume ratio; FA, fractional anisotropy; FDG, fluorodeoxyglucose; fMRI, functional MRI; GABA_A_, gamma-aminobutyric acid type A receptor; Glu, glutamate; Glx, glutamate + glutamine; Ki, influx constant; MD, mean diffusivity; mGluR5, metabotropic glutamate receptor 5; mI, myo-inositol; MRS, magnetic resonance spectroscopy; mSUV, modified standardized uptake value; NAA, N-acetylaspartate; PET, positron emission tomography; PFC, prefrontal cortex; SERT, serotonin transporter; VBM, voxel-based morphometry; VMAT2, vesicular monoamine transporter 2; VS, ventral striatum; tCr, total creatine; V_T_, total distribution volume. Timeframe: d, days; w, weeks; m, months; y, years.

Longitudinal studies have characterized trajectories of injury and recovery with abstinence. DBM shows frontal and temporal atrophy at the onset of abstinence, followed by volume gains over the next 6–9 months; gains are larger in individuals who maintain sobriety than in those who relapse, and gray-matter volume during the first week of abstinence can predict subsequent recovery (6, 42, 43). A global volumetric study similarly indicated that recovery is fastest during the first month of abstinence and then slows, whereas resumption of drinking is followed by rapid volume loss, underscoring the dynamic nature of these changes (6). From a network perspective, the recovery pattern identified by DBM involves regions of the fronto–ponto–cerebellar circuit, in line with the preferential involvement of this pathway described in chronic alcoholism. Historically, CT already demonstrated reversible atrophy after abstinence, an observation refined by modern MRI and morphometry, which reveal regional changes and sensitivity to abstinence versus relapse (44). Overall, chronic alcohol use is associated with diffuse damage predominating in frontal and cerebellar regions, callosal thinning and ventriculomegaly, accompanied by partial recovery that is faster early in abstinence and reversed by relapse; longitudinal DBM and volumetry provide sensitive markers to monitor these changes and to distinguish sustained abstinence from continued exposure.

Emerging evidence challenges the prevailing public perception that “moderate” alcohol consumption is harmless to the brain. In a 30-year longitudinal cohort, higher weekly alcohol intake was associated in a dose-dependent manner with greater likelihood of hippocampal atrophy, with no evidence of a protective effect of light drinking. Intake of only 14–21 units per week was linked to significant reduction of right hippocampal volume, as well as microstructural alterations in the corpus callosum and faster decline in lexical fluency, despite no group differences on other cognitive tests at follow-up (45). In the same direction, a multimodal analysis of the UK Biobank (36,678 participants) showed negative associations between alcohol quantity and global gray- and white-matter volume, as well as white-matter microstructure. These associations were widespread—with prominent effects in frontal, parietal and temporal cortices, cingulate, insula, brainstem, putamen and amygdala—and were already detectable with average intakes of only 1–2 units per day (46).

### 4.2 White matter microstructure and structural connectivity

Diffusion tensor imaging (DTI) studies in chronic drinkers consistently reveal microstructural compromise of white matter even when conventional MRI does not show overt volume loss. At a global level, reduced fractional anisotropy and increased mean diffusivity are observed, with a pattern that prominently affects anterior fibers and frontal integration tracts, including the corpus callosum, frontal forceps, internal and external capsules, fornix, and the cingulum and superior longitudinal fasciculi (2, 47). These abnormalities extend to frontolimbic, frontoparietal, fronto-occipital, corticostriatal and corticopontine pathways, supporting the notion of disconnection within networks that sustain executive control, motivation and habit formation (2).

Clinically and cognitively, DTI alterations are associated with memory decline and executive dysfunction. Aging may exacerbate these deficits, and several series report more pronounced microstructural abnormalities in women despite lower cumulative intake, suggesting greater biological susceptibility (2, 48). Abstinence has been associated with partial recovery of microstructure. Normalization of white matter microstructure has been described after as little as one month of abstinence, particularly in non-smokers, and callosal microstructural abnormalities can show recovery after a year of sobriety, although some deficits may persist despite sustained abstinence (49, 50). The topographic pattern, with anterior and frontocerebellar/mesencephalic predominance, is consistent with involvement of motivational and control systems. Disconnection of frontal–limbic tracts likely contributes to impulsivity, craving and behavioral rigidity, whereas mesencephalic and pontocerebellar involvement relates to psychomotor slowing and balance disturbances.

### 4.3 Task-based fMRI and cue reactivity

Alcohol-related cues (words, images, tastes or odors) reliably activate mesocorticolimbic circuitry in individuals with AUD. A meta-analysis of 28 studies (679 cases) showed robust activation of ventral striatum, ACC and ventromedial prefrontal cortex in response to alcohol cues, along with greater activation than controls in posterior cingulate, precuneus and superior temporal gyrus. Ventral striatal activation was the finding most consistently correlated with behavioral measures and the most likely to decrease following interventions (14).

In abstinent individuals, activation of striatum, ACC and medial prefrontal cortex during cue exposure predicts subsequent drinking during follow-up, whereas craving severity, prior consumption and duration of abstinence do not, suggesting that neural signals capture motivational processes that are not always accessible to self-report (13). Chronic alcohol use has also been associated with a shift from ventral, reward-related striatal engagement toward more dorsal “habit” circuitry, in line with a transition from goal-directed use to more automatic, stimulus–response patterns (2, 51). Cue reactivity in AUD has also been used as a pharmacodynamic marker. Trials with the opioid antagonist naltrexone and with the 5-HT3 receptor antagonist ondansetron have shown reduced ventral striatal activation to alcohol cues, and a single dose of amisulpride (a D2/3 receptor antagonist) decreased thalamic cue reactivity in abstinent subjects, supporting the usefulness of fMRI for monitoring therapeutic response (3, 52).

Task-based fMRI studies using cognitive paradigms show alterations in control and integration domains relevant for maintaining alcohol use. Recent reviews highlight changes in BOLD signal in executive-control regions (medial and dlPFC, ACC) and salience regions (insula) during tasks demanding self-control, working memory and decision-making, consistent with a reorganization of decision and control circuits in AUD (2, 3, 15). In inhibition (Go/No-Go, Stop-Signal) and interference (Stroop) tasks, multiple studies report abnormal prefrontal activation patterns in AUD, including reduced recruitment or inefficient engagement of dlPFC and ACC during response inhibition (53, 54). The prospective association between cue-elicited activation and relapse in abstinent patients, together with the sensitivity of this activation to pharmacological interventions, suggests that task-based fMRI may serve as a useful bridge between neurobiology and prognosis/treatment monitoring in AUD.

### 4.4 Resting-state functional connectivity

Resting-state fMRI studies consistently indicate altered functional network organization in heavy drinkers and individuals with AUD, with weakened within- and between-network connectivity, including the DMN (2). Voxel-wise functional connectivity density mapping further shows reduced connectivity in thalamus and cortical regions (visual, prefrontal, posterior cingulate and precuneus), alongside increased cerebellar connectivity (55). Within the DMN, a reorganization of connectivity hubs has been reported, with a shift from posterior/mid-cingulate dominance toward midbrain–mid-cingulate nodes, which has been interpreted as a marker of compromised executive control (2). Regional homogeneity analyses converge with these findings, reporting higher local synchrony in regions such as the superior/medial frontal gyrus and middle temporal gyrus in AUD (56). Building on these network-level alterations, resting-state functional connectivity features have also been used to differentiate AUD from controls using machine-learning classifiers, with discriminative connections spanning visual, sensorimotor, executive-control, reward and salience networks (57).

In abstinent subjects who dropped out after detoxification (compared with those who completed treatment), resting-state connectivity between striatum and insula, between the executive control network and the amygdala, and between the basal ganglia/salience network and the striatum, precuneus and insula is increased and correlates positively with craving intensity (58). In heavy drinkers, lower thalamic connectivity density has been associated with poorer cognitive performance (55). The relative increase in cerebellar connectivity density in heavy drinkers may represent a compensatory mechanism to sustain motor—and partly cognitive—performance in the face of cortical and thalamic deficits (55). Overall, resting-state fMRI suggests that chronic alcohol use shifts the balance of functional architecture away from control and self-referential networks (DMN/dorsal frontoparietal) toward salience and reward networks, with downstream effects on impulsivity, craving and executive control. Because resting-state metrics are relatively less affected by the acute vasomotor and substrate-shift effects of ethanol, they complement perfusion and metabolic imaging and help delineate intrinsic neuronal communication changes in AUD.

### 4.5 Cerebral blood flow and metabolism

In chronic drinkers and patients with AUD, perfusion and metabolic studies converge on a pattern of preferential dysfunction in frontolimbic and diencephalic networks, with additional cerebellar involvement. The severity and duration of exposure correlate with hypoperfusion and hypometabolism, which in turn are associated with executive, attentional and memory deficits (59).

ASL studies have documented prefrontal hypoperfusion (dlPFC, OFC and ACC), variably extending to temporal and parietal regions. These reductions correlate with poorer performance on tasks of inhibitory control, decision-making and working memory, and tend to be more pronounced in individuals with heavier or earlier-onset drinking (59, 60). SPECT studies have shown chronic hypoperfusion in frontal lobes, limbic structures, ACC and thalamus, even in patients without overt neurological symptoms, and this pattern has been linked to episodic memory impairment and emotional disturbances (59, 61, 62). Figure 1 illustrates persistent diffuse cortical hypoperfusion (left-predominant) despite 18 months of abstinence in a long-term alcohol and cocaine user presenting with cognitive complaints.

**Figure 1:**
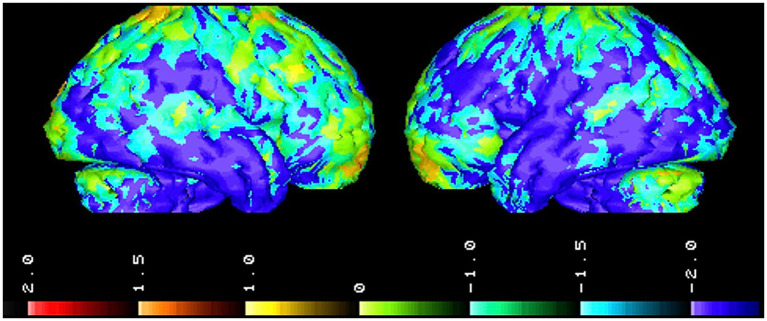
Cerebral perfusion SPECT in a 40-year-old man with more than 20 years of heavy alcohol use and cocaine consumption. The patient had been abstinent for 18 months and presented with moderate memory, attentional, and dysexecutive symptoms. Surface-projected statistical maps (lateral views) show diffuse cortical hypoperfusion, more pronounced in the left hemisphere, compared with age-matched healthy controls. The color scale indicates the magnitude of perfusion differences expressed in standard deviations from the control mean (negative values denote reduced perfusion). Overall, this pattern supports persistent functional abnormalities and is consistent with long-term sequelae associated with chronic substance use.

FDG-PET has consistently demonstrated frontal and parietal hypometabolism in chronic drinkers and in recently abstinent patients, with robust associations to executive and attentional deficits (59, 63). In some clinical profiles, additional reductions are seen in cingulate, insula and thalamus, and when cognitive impairment is more severe the dysfunction becomes more widespread, involving medial networks and cerebellar circuits (3, 59, 64). Recent FDG-PET evidence further links executive dysfunction in AUD to regional hypometabolism in cingulate-frontal networks (65). It has been proposed that chronic prefrontal dysfunction weakens top–down modulation of reward and habit circuits, thereby favoring compulsive consumption and relapse. Within this framework, the partial uncoupling of flow and metabolism observed during acute intoxication tends to evolve into a stable profile of chronic hypoperfusion and hypometabolism in vulnerable networks (16, 59, 64).

ASL has also proven useful for monitoring changes with abstinence, showing partial increases in frontal and parietal gray matter perfusion over the first weeks (66). Longitudinal FDG-PET studies document partial recovery of cortical metabolism during the first 2–4 weeks of abstinence, without complete normalization in all cases; residual frontal deficits have been associated with more frequent relapses and poorer neuropsychological performance (59, 67). Thus, metabolic–perfusion normalization may be incomplete and may coexist with persistent structural damage.

### 4.6 Molecular imaging

#### 4.6.1 Dopamine

PET and SPECT studies have consistently characterized dopaminergic alterations in AUD as summarized in a recent dedicated review (68). Striatal D2/3 receptor availability is typically reduced in recently detoxified individuals with AUD, supporting attenuated postsynaptic dopaminergic signaling in mesostriatal circuits critical for reward processing and inhibitory control (16, 69). Quantitatively, ^11^C-raclopride studies report approximately 20% lower striatal D2/3 availability (including ventral and dorsal striatum) in AUD compared with controls (69). The magnitude of these reductions may vary with abstinence duration, which is likely an important contributor to between-study heterogeneity (68).

Dopaminergic reactivity to pharmacological or alcohol challenges is also diminished. Amphetamine challenge in detoxified patients reveals markedly blunted dopamine release in the striatum, accompanied by abnormal functional coupling between striatum and prefrontal cortex, consistent with impaired cortical regulation of mesolimbic dopaminergic activity (70). This pattern may contribute both to a bias toward alcohol-related cues and to reduced sensitivity to non–drug-related rewards.

Presynaptic markers refine this picture. In detoxified patients, one study found no group differences in striatal ^18^F-DOPA uptake, but lower uptake—particularly in ventral striatum—correlated with greater craving and relapse risk (71). In the same line, craving has been inversely related to D2/3 availability (72). These relationships are illustrated in Figure 2.

**Figure 2:**
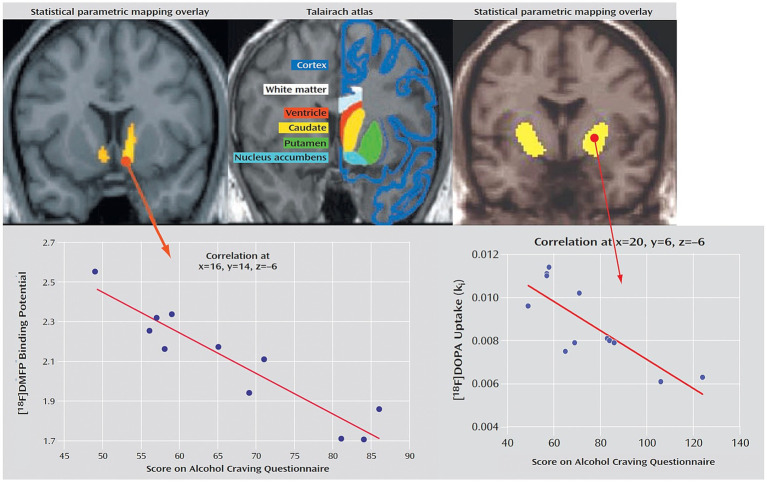
Relationship between alcohol craving and striatal dopaminergic markers during early abstinence. Adapted with permission from Heinz et al. (71, 72). Statistical parametric mapping (SPM) coronal PET correlation maps depict negative associations between acute alcohol craving (Alcohol Craving Questionnaire) and (i) dopamine D2 receptor availability assessed with ^18^F-DMFP (binding potential; upper left panel and lower left scatterplot), and (ii) presynaptic dopamine synthesis capacity assessed with ^18^F-DOPA (net influx constant K_i_/net blood–brain clearance; upper right panel and lower right scatterplot). In detoxified alcohol-dependent patients, D2 receptor availability in the ventral striatum/NAc was inversely correlated with craving after 3 weeks of abstinence (*n* = 11), and ^18^F-DOPA K_i_ in the bilateral striatum (including putamen) was inversely correlated with craving after 5 weeks of abstinence (*n* = 12); no significant correlations were observed in healthy comparison subjects. For display purposes, significance thresholds of *p* < 0.001 (D2 study) and *p* < 0.01 (^18^F-DOPA study) were applied. The central panel illustrates the anatomical correspondence of the significant clusters by comparison with the Talairach and Tournoux atlas. Arrows indicate the voxel/cluster used for the scatterplots (Talairach coordinates shown in each panel).

High-affinity D2/3 ligands such as ^18^F-fallypride have allowed assessment of both striatal and extrastriatal regions. In recently abstinent individuals with AUD, these studies confirmed reduced striatal D2/3 availability and identified abnormal extrastriatal binding patterns (e.g., in thalamus). In a subgroup with prolonged abstinence or marked reduction in consumption, follow-up imaging showed increases in binding potential in striatum and thalamus at one year compared with early measurements, with larger gains in those who remained fully abstinent than in those who only reduced drinking—suggesting partial reversibility of D2/3 availability proportional to clinical improvement (73).

Presynaptic integrity has also been examined with tracers targeting the vesicular monoamine transporter type 2 (VMAT2), such as ^11^C-DTBZ. In a small series of seven men with severe AUD, decreased ^11^C-DTBZ binding in caudate and putamen indicates a loss of nigrostriatal monoaminergic terminals and vesicular dopamine stores, a deficit that likely contributes to the reduced stimulant-induced dopamine release observed in challenge paradigms (74).

Beyond D2/3 and presynaptic markers, convergent post-mortem and animal data indicate that striatal D1 receptors and dopamine transporters undergo dynamic regulation across the addiction–abstinence cycle, with marked changes in D1/DAT binding in subjects with AUD and in rodent models of chronic exposure and withdrawal (75). In parallel, these data suggest that D1 receptor–related signaling is altered during protracted abstinence, consistent with a potential contribution of D1-mediated striatal mechanisms to alcohol-related phenotypes (75). Together, these findings point to D1 receptors as a potentially relevant, but still underexplored, target for future molecular imaging studies in AUD.

Taken together, available pre- and postsynaptic findings are broadly consistent with a chronic mesolimbic dopaminergic deficit, most prominent in ventral striatum, together with altered prefrontal modulation of reward and salience processing (16). Across studies, this pattern has been linked to craving, impulsivity and reduced sensitivity to natural rewards, and may contribute, along with alterations in other systems (opioid, glutamatergic), to dysfunction within salience and executive-control circuits. In this framework, reduced D2/3 availability and blunted dopaminergic reactivity can be interpreted as candidate mechanisms underlying compulsive alcohol use and the imbalance between habit-related responding and prefrontal control.

Regarding the effects of abstinence, longitudinal evidence suggests slow and incomplete recovery. In one series of 14 patients with AUD studied with ^11^C-raclopride after 6 weeks of abstinence and again 1–4 months later, striatal D2/3 availability remained reduced and did not change significantly over time (76). In contrast, in a smaller subgroup followed for one year with ^18^F-fallypride, striatal and thalamic binding increased by approximately 30%, with larger improvements in the two individuals who maintained complete abstinence compared with the two who only reduced their drinking—compatible with partial normalization of D2/3 availability in proportion to clinical recovery, although sample size and heterogeneity preclude firm conclusions (73).

#### 4.6.2 Opioid, GABA, serotonin, glutamate and endocannabinoid systems

In chronic alcohol users, *μ*-opioid receptor PET with ^11^C-carfentanil reveals a heterogeneous pattern, with some series reporting increased binding in ventral striatum and others decreased binding in frontal and parietal cortices (16). Regionally, *μ*-opioid receptor availability in reward-related regions, particularly the ventral striatum/NAc, correlates positively with alcohol craving (77). Because ^11^C-carfentanil binding is sensitive to endogenous opioid tone, apparent increases in binding may reflect lower occupancy by endogenous opioids rather than higher receptor density. This interpretation is supported by post-mortem findings of marked *μ*-receptor loss in striatum in severe alcoholism (78). Overall, converging PET and post-mortem evidence suggests an altered opioid state in AUD, likely characterized by μ-receptor loss with regionally increased apparent binding due to reduced endogenous tone; this provides a biological rationale for using opioid antagonists (naltrexone/nalmefene) to prevent relapse.

Regarding the GABA system (benzodiazepine-sensitive GABA_A_ receptors), SPECT/PET studies using non-selective ligands (flumazenil/iomazenil) most consistently report approximately 6–20% decreases in binding in frontal cortex, ACC and cerebellum, while findings in other regions are more variable, likely reflecting methodological and sample heterogeneity, including differences in abstinence duration (9, 79). Using the α5-selective ligand ^11^C-Ro15-4513, reductions were observed in NAc and right hippocampus after 6 or more weeks of abstinence, suggesting a specific limbic component (80). Pharmacological challenge paradigms with ^11^C-flumazenil PET and midazolam have shown that, despite similar benzodiazepine receptor occupancy, alcohol-dependent individuals exhibit markedly shorter EEG-defined sleep time than controls, consistent with reduced GABA_A_ sensitivity (9). FDG-PET studies using lorazepam have shown that benzodiazepine-induced decrements in glucose metabolism are attenuated in alcohol-dependent individuals in the thalamus, basal ganglia and OFC compared with controls (81). OFC abnormalities may persist into later stages of detoxification (approximately 8–11 weeks post-detox), with a trend toward a blunted lorazepam response, consistent with residual alterations in inhibitory (GABAergic) modulation within frontostriatal circuits (82). Longitudinally, a transient increase in GABA_A_ availability has been described during the first week of abstinence, normalizing by week 4 (83). In contrast, cross-sectional PET/SPECT studies conducted after several weeks to months of abstinence have reported lower GABA_A_ binding compared with controls, suggesting that relative reductions may predominate in later stages of abstinence (64).

Serotonin transporter (SERT) availability in brainstem appears reduced by approximately 30%, correlating with cumulative alcohol exposure and with depressive/anxious symptoms during early abstinence—clinical features that increase relapse risk (84). Genetic variation in 5-HTT may modulate these SERT changes and associated negative mood states, and chronic excessive alcohol intake has been proposed to exert neurotoxic effects on brainstem serotonergic neurons and projections in long-standing AUD (8).

Glutamatergic findings are more limited. In non-smoking men with AUD and a minimum of 25 days of abstinence, PET with ^11^C-ABP688 demonstrated increased availability of metabotropic glutamate receptor 5 (mGluR5) in amygdala and temporal lobe, associated with lower temptation to drink, implicating this receptor in the affective and motivational processing of alcohol-related cues (85).

Endocannabinoid CB1 receptor PET with tracers such as ^18^F-FMPEP-d2 and ^18^F-MK-9470 has shown a generalized 15–30% reduction in CB1 availability in AUD, correlating with years of drinking and persisting after four weeks of abstinence (86, 87). Given that CB1 receptors are located presynaptically on GABAergic interneurons and glutamatergic terminals, these findings position the endocannabinoid system as a key modulator of salience and stress with potential impact on craving and relapse.

Collectively, alterations in opioid, GABAergic, serotonergic, glutamatergic and endocannabinoid systems, together with reduced mesolimbic dopaminergic signaling, converge on limbic and prefrontal circuits involved in reward, salience and control. This integrative framework helps explain craving, impulsivity and relapse in AUD, and guides the development of pharmacological interventions targeting specific systems and their interactions.

#### 4.6.3 Neuroimmune and synaptic PET imaging

Human TSPO PET studies in AUD have not shown a consistent increase in TSPO signal. In recently detoxified patients, ^11^C-PBR28 PET has shown lower global binding in one study and reduced hippocampal uptake in another (88, 89). A third study found no overall difference between AUD and controls but reported lower ^11^C-PBR28 binding in medium-affinity binders; it also found that higher plasma cholesterol was associated with lower tracer binding, raising the possibility that endogenous ligands may influence TSPO PET estimates in AUD (90). Preclinical findings have further illustrated the complexity of TSPO imaging in chronic alcohol exposure: in alcohol-dependent rats, increased TSPO binding on *in vitro* autoradiography was found in the thalamus and hippocampus despite unchanged *in vivo* ^11^C-PBR28 PET signal, indicating that TSPO abnormalities in chronic alcohol models are method-dependent and not uniformly reproduced by in vivo PET (91). Overall, the available evidence suggests that TSPO PET abnormalities in AUD reflect complex and stage-dependent glial or neuroimmune alterations rather than a uniform increase in neuroinflammatory signal (92).

Synaptic density imaging with SV2A PET provides an additional molecular window into chronic alcohol-related brain changes. In a recent ^11^C-UCB-J PET study, individuals with AUD showed lower SV2A binding in the frontal cortex, striatum, and hippocampus, with a similar trend in the cerebellum, consistent with reduced synaptic density in circuits implicated in executive control, reward processing, and memory (93). Within the AUD group, lower SV2A binding was also associated with greater drinking severity. Although still limited to an early clinical literature, these findings suggest that chronic alcohol use may be associated with reduced synaptic density *in vivo* and identify synaptic integrity as a promising target for future longitudinal and treatment studies.

### 4.7 Magnetic resonance spectroscopy

In chronic alcohol use, proton MRS studies have relatively consistently reported lower NAA and choline-related metabolite levels in frontal lobe, medial temporal structures, cerebellum and thalamus, findings interpreted as markers of neuronal compromise and membrane alterations (1–3, 94–96). Several longitudinal series show improvement with abstinence, particularly in cerebellar vermis and frontal cortex over 3–6 months, although shorter follow-up studies do not always detect changes, suggesting a slow recovery process (95, 97).

With respect to glutamate, heavier drinkers exhibit lower Glu levels in frontal white matter than light drinkers (98). During very early abstinence/withdrawal, elevated Glu/Glx in ACC and increased Glu in the NAc have been reported, correlating with craving and trending toward normalization by approximately two weeks (99–101). In subsequent weeks, Glu has been described as reduced in ACC, with partial increases toward normalization over subsequent weeks (102). At later stages of abstinence, some MRS studies report lower Glu/Glx levels in ACC and other cortical regions relative to controls, although results vary across samples and time windows (1, 2). Consistent with this variability, a recent meta-analysis did not identify significant overall differences in glutamate-related metabolites between alcohol misuse/AUD groups and controls (96). However, conventional MRS cannot distinguish intracellular from extracellular glutamate pools, complicating interpretation (2). Nevertheless, longitudinal data are compatible with an initial hyperexcitable state—potentially potentiating craving and relapse vulnerability—, followed by a relative hypoexcitable phase that may contribute to apathy, anergia and cognitive deficits.

In addition, increased myo-inositol has been observed in the thalamus and ACC during early detoxification, consistent with glial changes, with a trend toward normalization in more prolonged abstinence (1, 103). Taken together, NAA, Cho and mI tend to recover with abstinence, supporting partial reversibility of the metabolic profile, whereas Glu/Glx shows more complex, abstinence-duration–dependent dynamics. In summary, MRS provides a tissue-level neurochemical perspective that situates macroscopic structural and functional abnormalities within changes in neuronal integrity, glial state and excitatory–inhibitory balance. By capturing dynamic metabolite shifts across withdrawal and abstinence, it may help distinguish more reversible from neurotoxic components of alcohol-related brain changes and offer stage-sensitive markers to monitor treatment response and clinical recovery.

## 5 Neuroimaging correlates of adolescent alcohol use

Adolescence represents a window of heightened vulnerability to the effects of alcohol on the brain. During this period, frontolimbic circuits supporting reward processing, cognitive control and emotion regulation are still maturing, with an imbalance between relatively earlier development of subcortical reward systems and the slower maturation of prefrontal control networks (10, 104). This network configuration may increase sensitivity to alcohol exposure during this stage of development. Within this context, binge drinking—typically defined as the consumption of ≥5 drinks in males or ≥4 in females on a single occasion, usually within about 2 h, resulting in a blood alcohol concentration ≥0.08 g/dL—has become a highly prevalent pattern among adolescents and emerging adults (105). Neuroimaging studies in these groups, summarized in recent systematic reviews, indicate that even in the absence of formal AUD diagnoses, this pattern is associated with measurable alterations in brain structure and function, particularly in frontoparietal and frontostriatal networks (10, 106). Neuroimaging correlates of alcohol use during adolescence are summarized in Table 3.

**Table 3:** Neuroimaging correlates of alcohol use during adolescence.

Modality	Specific technique (metric)	Key region/network (effect direction)	Exposure/timeframe	Comment	References
Structural MRI	VBM/cortical thickness (volume/thickness)	PFC/OFC, ACC, temporal cortex, cerebellum (GM volume and/or thickness altered, often ↓)	Initiation to escalation; binge/heavy drinking (m–y)	Deviations in maturational trajectories; dose–response reported	(10, 106, 118)
	Longitudinal morphometry (growth trajectories)	WM volume growth attenuated; regionally frontal/callosum (↓ relative age-related increase)	Prospective cohorts (y)	Deviations from normative cortical/WM growth trajectories after drinking onset; caution with comorbidities	(10, 116, 118)
DTI	FA/MD (WM microstructure)	Corpus callosum and major association/projection tracts (FA ↓ and/or diffusivity ↑)	Heavier use and higher peak exposure (m–y)	Poorer white matter integrity; confounding by nicotine/cannabis common	(114, 115)
Task fMRI	Inhibitory control/working memory (BOLD)	Frontoparietal and frontostriatal control networks (altered recruitment; dlPFC/dACC effects)	During tasks; associated with binge/heavy patterns (m–y)	Altered recruitment of control networks. May reflect vulnerability and/or alcohol-related disruption.	(109, 110)
	Reward processing (BOLD)	Striatum, limbic regions (altered reward responsivity; mixed direction)	Initiation and escalation (m–y)	Perturbed integration of motivational and cognitive signals	(112, 113)

ACC, anterior cingulate cortex; AUD, alcohol use disorder; BOLD, blood oxygen level-dependent; dACC, dorsal anterior cingulate cortex; dlPFC, dorsolateral prefrontal cortex; DTI, diffusion tensor imaging; FA, fractional anisotropy; fMRI, functional MRI; GM, gray matter; MD, mean diffusivity; OFC, orbitofrontal cortex; PFC, prefrontal cortex; VBM, voxel-based morphometry; WM, white matter; m, months; y, years.

Longitudinal MRI and fMRI studies suggest that the initiation and escalation of alcohol use during adolescence are linked to altered neurodevelopmental trajectories. Compared with non-drinking peers, adolescent and emerging adult binge drinkers show deviations in age-related change of cortical thickness and white-matter maturation, together with altered functional responses during cognitive and reward tasks (107–110). In working-memory paradigms, adolescents who initiate heavy (binge) drinking show greater recruitment of frontal and parietal regions than controls, a pattern often interpreted as increased neural effort or functional compensation (111). During reward-related decision making, altered activation has been described in dorsal striatum in adolescent binge drinkers, and ventral striatal responses during risk–reward decisions have been associated with earlier onset of binge drinking; together, these findings suggest disrupted maturation of circuits integrating motivational and cognitive signals (112, 113).

At the microstructural level, DTI studies in adolescent drinkers most consistently report poorer white matter integrity (including reduced FA and/or increased diffusivity) in major association and projection tracts with frontal connections (e.g., superior longitudinal fasciculus, corona radiata, thalamic radiations), which relates to heavier alcohol use and higher peak alcohol exposure (114, 115).

In addition, the normative increase in white-matter volume and integrity across adolescence appears attenuated in binge drinkers compared with non-drinking peers (10, 97, 116, 117). These effects show dose–response relationships and, in some cohorts, are modulated by concomitant cannabis use, emphasizing the need to carefully control for polysubstance use when interpreting white-matter findings (10, 116, 118).

From a volumetric perspective, several studies report that adolescent and emerging adult drinkers exhibit reductions not only in frontal regions but also in ACC, temporal and cerebellar structures, often with sex-specific patterns (10, 106, 116, 117). In a carefully selected cohort of adolescents with AUD but without psychiatric or other substance-use comorbidities, gray-matter density was lower in a large left lateral frontal–temporal–parietal cluster, accompanied by sex-dependent differences in thalamic and putaminal volumes, whereas hippocampal volumes did not differ significantly between AUD and control groups (118). Taken together with work in broader age ranges, these findings suggest that hippocampal involvement may depend on both developmental stage and clinical severity, with smaller and less consistent effects in adolescents than in adults (10, 106, 118).

Overall, prospective and cross-sectional findings suggest a temporal direction in which alcohol use during adolescence contributes to deviations in maturational trajectories—accelerating or exaggerating cortical thinning in some regions, blunting expected gains in white-matter integrity and altering the tuning of reward and control networks—rather than merely reflecting pre-existing vulnerability. These brain alterations are associated with differences in attention, executive functions and impulsivity and with greater propensity to maintain or escalate binge drinking. In this sense, adolescent neuroimaging contributes both to characterizing early neural signatures of risk and to understanding how a pattern often perceived as “normative” (binge drinking in youth) can contribute to long-term vulnerability to AUD.

## 6 Sex differences in alcohol-related brain alterations

Sex is increasingly recognized as a critical biological variable in the neurobiology of AUD, although it remains insufficiently addressed in neuroimaging studies. Historically, most studies have been conducted in predominantly male samples, and even when women are included, sex-specific analyses are often underpowered or inconsistently reported (119). As a result, evidence suggests sex-related differences across neurochemical, structural, and functional domains, although findings remain heterogeneous and modality-dependent.

From a structural perspective, MRI studies indicate that alcohol-related brain alterations may differ between men and women. Early work demonstrated sex-by-diagnosis interactions, with abnormalities in cortical white matter and sulcal volumes primarily observed in men with AUD, whereas findings in women were less consistent; alcohol-related changes also interacted with age in both sexes, and white matter volumes in women were associated with duration of abstinence (120). More recent large-scale studies provide a more nuanced view, showing both shared and sex-modulated effects. For example, ENIGMA analyses have shown reduced hippocampal volumes in both men and women, whereas amygdala subregions appear more affected in men and may scale with alcohol exposure (121). Whole-brain voxel-based analyses similarly indicate that structural abnormalities are present in both sexes but differ in regional expression, with greater cerebellar and white matter involvement in women and more localized cortical effects in men (122).

Diffusion MRI studies further illustrate the complexity of sex effects on white matter microstructure. Some investigations report sex-by-diagnosis interactions, with reduced FA in men with AUD and preserved or higher FA in women (123), whereas others find widespread abnormalities without significant sex interactions (124). Age appears to be an important modifier, with more pronounced age-related alterations in AUD (124).

Functional MRI studies also indicate sex-related differences in large-scale brain networks. In abstinent individuals, men show greater activation and stronger connectivity to aversive stimuli, whereas women show reduced activation and weaker connectivity relative to controls (125). More recent work examining stress- and cue-induced responses suggests differential involvement of cortico–striatal–limbic circuits, with distinct associations between neural responses and future drinking outcomes in men and women (126). However, findings on sex differences in alcohol cue reactivity have not been consistent (127).

FDG-PET studies provide complementary systems-level evidence. Acute alcohol administration produces greater reductions in cerebral glucose metabolism in men than in women, indicating a blunted metabolic response in females (128). In abstinent individuals, men more often show group-level reductions in brain metabolism and structure, whereas findings in women are more variable and more closely related to alcohol exposure or clinical factors (129).

At the neurochemical level, PET studies remain limited but suggest that sex modulates dopaminergic and opioid responses. Alcohol-induced dopamine release in the ventral striatum has been reported to be greater in men and to correlate with reinforcing effects primarily in men (119, 130), although broader evidence indicates mixed findings depending on experimental conditions (131). Similarly, sex differences in opioid receptor availability have been reported, but findings remain inconsistent and often underpowered (119).

Taken together, multimodal neuroimaging evidence suggests that sex influences alcohol-related brain alterations across structural, functional, metabolic, and neurochemical domains. Although findings vary across modalities and study designs, the literature supports that sex may modulate large-scale brain systems implicated in AUD—particularly reward, salience/stress, and executive control networks—rather than producing uniform regional effects. These influences interact with age, alcohol exposure, abstinence, and clinical state. Future multimodal studies incorporating sex as a primary analytic variable will be essential to refine sex-informed models of vulnerability, progression, and treatment response in AUD.

## 7 Alcohol-related neurological syndromes

Beyond the more subtle, network-level changes described above, chronic alcohol use is also associated with a series of well-defined neurological syndromes. These conditions reflect the combined impact of alcohol toxicity, nutritional deficiencies (particularly thiamine), liver disease, osmotic shifts and withdrawal, and they exhibit relatively characteristic neuroimaging patterns that can guide diagnosis and management (1, 132).

The best-characterized entities are Wernicke encephalopathy (WE) and Korsakoff syndrome (KS). WE is an acute encephalopathy due to thiamine deficiency that typically manifests with a triad of confusion, gait and balance disturbance, and eye movement abnormalities. If not treated, this condition can progress to KS, a chronic amnestic disorder marked by severe anterograde memory loss and frequent confabulation. On MRI, acute WE usually presents with bilateral T2/FLAIR signal increase in the medial thalami, mammillary bodies, periaqueductal gray and tectal plate, occasionally accompanied by contrast enhancement (7, 132, 133). In contrast, KS is more often characterized by atrophy of the mammillary bodies, thalamus and hippocampus, together with marked global atrophy exceeding that observed in uncomplicated chronic alcohol use (7, 132, 133). In younger adults, Wernicke–Korsakoff syndrome can occasionally present with normal structural MRI, while perfusion SPECT may demonstrate thalamic and frontotemporal hypoperfusion, suggesting added sensitivity of functional techniques in atypical or early presentations (134). These perfusion abnormalities are illustrated in Figure 3. Early recognition of these patterns is critical, as parenteral thiamine can prevent progression to irreversible deficits if administered promptly (7, 132).

**Figure 3:**
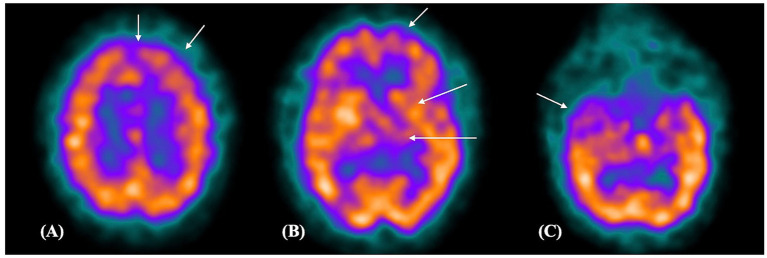
Cerebral perfusion SPECT in a young man with Korsakoff syndrome. Selected axial slices demonstrate hypoperfusion involving the prefrontal cortex (left-predominant; panels **A,B**), thalamus (markedly left-predominant; panel **B**), left striatum **(B)**, and right anterior temporal cortex **(C)** (arrows).

Other structural syndromes related to chronic alcohol use include hepatic encephalopathy, osmotic demyelination syndrome, Marchiafava–Bignami disease, alcoholic cerebellar degeneration and alcohol-related dementia (1, 132). In hepatic encephalopathy secondary to advanced liver disease, MRI often shows T1 hyperintensity of the globus pallidus (and sometimes substantia nigra) due to manganese deposition, along with white-matter signal changes that may improve when liver function is restored (132, 135, 136). Osmotic demyelination, typically triggered by rapid correction of chronic hyponatremia in malnourished or alcohol-dependent patients, manifests as central pontine myelinolysis and extrapontine lesions in basal ganglia, thalamus and cortex (132, 137). Marchiafava–Bignami disease is characterized by demyelination and necrosis of the corpus callosum, with T1 hypointensity and T2/FLAIR hyperintensity of the genu and/or splenium and possible extension into adjacent white matter (132, 138). Alcohol-related cerebellar degeneration preferentially affects the anterior–superior vermis, while alcohol-related dementia presents as a frontal–subcortical cognitive syndrome with predominant frontal atrophy and ventricular enlargement, sometimes showing partial reversibility with long-term abstinence (1, 132).

These classical alcohol-related neurological syndromes occur predominantly in the setting of severe and prolonged AUD, often in association with nutritional deficiency, liver disease, or other medical complications, and can be viewed as macroscopic, structurally overt endpoints along the same continuum of brain vulnerability described in earlier sections. Their characteristic imaging signatures, together with their potential for partial reversibility if treated promptly, highlight the role of neuroimaging not only in delineating mechanisms and biomarkers, but also in enabling timely clinical interventions.

## 8 Biomarkers of vulnerability and prognosis

### 8.1 Trait and state markers

Vulnerability to AUD can be captured with trait markers—endophenotypes present before the onset of problematic use—and with state markers, which reflect the current impact of alcohol exposure and its partial reversibility with abstinence. Trait markers are particularly relevant for identifying individuals at risk (e.g., family history of AUD, low subjective response to alcohol, specific genetic variants), whereas state markers can inform prognosis and track change during treatment and recovery.

Recent frameworks integrating behavioral, circuit-level and molecular domains emphasize that alcohol use and AUD emerge from interactions between pre-existing neurobiological vulnerabilities and alcohol-induced neuroadaptations within large-scale networks (2, 15). Within this perspective, neuroimaging does not operate in isolation but contributes mechanistic measures of function and chemistry in circuits of reward, salience, negative affect and cognitive control, which can be combined with clinical, genetic and behavioral data to refine risk stratification and prognosis (2, 11, 15).

In the following sections, we discuss imaging-linked associations with familial risk and specific genetic variants—including candidate single-nucleotide polymorphisms and other polymorphisms—that have been linked to alcohol-related phenotypes and intermediate neural markers of vulnerability. These findings should be interpreted cautiously, however, as many candidate-gene studies were conducted in relatively small samples, may be ancestry-specific, and have shown variable replication (139). More broadly, recent genetic work has also highlighted the relevance of polygenic risk scores, which capture the cumulative contribution of many loci with individually small effects and have shown associations with alcohol-related behaviors and symptom dimensions (139, 140). This caveat is particularly relevant when interpreting the imaging-genetics findings summarized below. Key neuroimaging biomarkers of vulnerability and prognosis are summarized in Table 4.

**Table 4:** Neuroimaging biomarkers of vulnerability and prognosis in alcohol use and AUD.

Modality	Specific technique (metric)	Key biomarker (region/network; effect direction)	Outcome predicted	Comment	References
ASL MRI	ASL Alcohol challenge (ΔCBF)	Frontal CBF response to alcohol (blunted ↑ in low responders)	Future heavier drinking; alcohol-related problems	Imaging correlate of the low subjective response phenotype (attenuated pharmacodynamic sensitivity)	(17)
Task fMRI	Intertemporal decision-making fMRI (BOLD)	Frontoparietal activation differences by COMT Val158Met (Val/Val vs. Met carriers)	Impulsive intermediate phenotype; clinical heterogeneity	COMT-related cortical dopamine tone may shape impulse-control circuitry relevant to AUD subtypes	(151)
	Emotion/reward processing tasks in early abstinence (BOLD)	vmPFC/ACC activation to relaxing stimulus (↑) and ventral striatal responses to positive stimuli (↓ vs. ↑ protective)	Relapse risk in early abstinence	Prefrontal–striatal balance: greater vmPFC/ACC engagement predicts higher relapse risk; preserved ventral striatal responsivity is protective	(15, 141, 142)
Task fMRI + genetics	Alcohol-cue fMRI (BOLD) + GABRA2 risk alleles	Medial frontal activation to alcohol cues (↑)	Vulnerability to heavy drinking/AUD	Variant may enhance reinforcing impact and salience of alcohol-related signals	(11, 153, 154)
	Monetary incentive delay task fMRI (BOLD) + GABRA2 rs279858	NAc activation to reward cues (↑ in G-allele carriers)	Alcohol-related problems (adolescents; high familial risk)	Striatal hyperreactivity mediates genotype–alcohol problems association	(11)
SPECT	DAT SPECT (^123^I-β-CIT; DAT availability)	Putaminal DAT availability (lower in 9/10-repeat vs. 10/10 DAT1 VNTR)	Vulnerability/clinical course (individual differences)	Alcoholism status per se not associated; genotype modulates presynaptic marker interpretation	(150)
PET	D2/3 PET (availability) + FDG-PET (metabolism)	Ventral striatum D2/3 availability (↑) + dlPFC/OFC/ACC metabolism (↑) in FH+	Resilience/reduced likelihood of transition to dependence	Proposed protective endophenotype supporting better inhibitory and emotional control	(143, 144)
	D2/3 PET (^18^F-fallypride) (Availability (BP_ND_))	Putaminal D2/3 availability shows a linear decrease (low-risk > high-risk > detoxified AUD)	Vulnerability (dimensional risk gradient)	Severity inversely related to striatal D2/3 availability; consistent with an intermediate (pre-AUD) risk marker	(145)
	Stimulant challenge dopamine-release PET (e.g., ^11^C-raclopride displacement)	Ventral striatal dopamine release (blunted in AUD)	Craving; impulsivity; worse clinical outcomes	Trait-like hypodopaminergic response in AUD; links to clinical severity	(16, 70, 147)
	Expectation/alcohol-cue paradigm dopamine-release PET	Ventral striatal dopamine release (potentiated by alcohol expectation; strongest in higher genetic risk)	Vulnerability phenotype (anticipatory cue responsivity)	Suggests exaggerated anticipatory dopaminergic response to alcohol-related cues in higher-risk individuals	(148, 149)
	μ-opioid PET (^11^C-carfentanil; alcohol-induced endogenous release)	Ventral striatum and OFC (greater alcohol-induced ↓ binding potential → greater endogenous opioid release)	Escalation risk; higher-risk drinking patterns	Heightened opioid hedonic sensitivity may represent a risk phenotype (stronger ‘high’ and reinforcement)	(16, 37)
PET + genetics	D2/3 PET (availability) + DRD2/ANKK1 Taq1A polymorphism	Lower striatal D2/3 availability (A1 allele carriers)	Higher AUD vulnerability	Taq1A A1 allele linked to reduced D2/3 receptor density.	(146)
	Dopamine-release PET + OPRM1 A118G genotype	Alcohol-induced striatal dopamine response (enhanced in Asp40 carriers)	Vulnerability and treatment response (debated)	Interindividual μ-opioid signaling may amplify reward-related dopamine responses; evidence limited	(152)
PET + MRS	^18^F-fallypride PET (D2/3) + ACC ^1^H-MRS (GABA)	Inhibitory–dopaminergic coupling (ACC GABA vs. associative striatal D2/3) disrupted in AUD and high-risk relatives	Vulnerability and familial risk	Decoupling may reflect impaired cortical regulation over mesolimbic dopamine signaling	(155)

ACC, anterior cingulate cortex; ANKK1, ankyrin repeat and kinase domain containing 1; AUD, alcohol use disorder; ASL, arterial spin labeling; Asp40, Asp40 variant (OPRM1 A118G); BOLD, blood oxygen level-dependent; CBF, cerebral blood flow; COMT, catechol-O-methyltransferase; DAT, dopamine transporter; DRD2, dopamine D2 receptor gene; dlPFC, dorsolateral prefrontal cortex; FDG, fluorodeoxyglucose; fMRI, functional MRI; FH+, positive family history; GABA, gamma-aminobutyric acid; GABRA2: GABA type A receptor subunit α2; MRS, magnetic resonance spectroscopy; NAc, nucleus accumbens; OFC, orbitofrontal cortex; OPRM1, μ-opioid receptor gene; PET, positron emission tomography; SPECT, single-photon emission computed tomography; vmPFC, ventromedial prefrontal cortex; VNTR, variable number tandem repeat.

### 8.2 Blood flow and functional activation markers

In early abstinence, patterns of task-related activation have been associated with relapse risk (15). Greater activation in ventromedial prefrontal cortex and ACC in response to a relaxing stimulus predicted a higher risk of relapse, potentially indexing heightened appraisal/regulatory engagement during low-demand states and diminished natural reward sensitivity, whereas stronger ventral striatal responses to positive stimuli were associated with a lower risk, consistent with preserved reward responsiveness as a protective factor (141, 142). This dissociation highlights a prefrontal–striatal balance that may influence relapse vulnerability and clinical outcome.

A particularly informative trait marker is the low subjective response to alcohol, a genetically influenced phenotype in which individuals require higher doses to experience intoxicating effects. ASL studies show that these “low responders” exhibit smaller alcohol-induced increases in frontal CBF for the same dose, consistent with attenuated pharmacodynamic sensitivity at the brain level (17). This phenotype is associated with future risk of heavier drinking and alcohol-related problems, and the blunted CBF response may represent an imaging correlate of this vulnerability (17).

### 8.3 Dopaminergic markers of risk and resilience

In contrast to the reduced striatal D2/3 receptor availability that characterizes established AUD, unaffected individuals with a positive family history of AUD can show increased D2/3 availability in ventral striatum together with higher metabolism in dlPFC, OFC and ACC (143). This profile has been interpreted as a potential protective endophenotype, associated with better inhibitory and emotional control and greater capacity to engage prefrontal regions during decision-making, thereby reducing the likelihood of transitioning to dependence (143, 144).

Extending this framework, an ^18^F-fallypride PET study supported a dimensional view of dopaminergic vulnerability by showing that dorsal striatal D2/3 receptor availability was lowest in detoxified AUD patients, highest in low-risk controls, and intermediate in individuals at high risk for AUD, with a significant linear decrease particularly in the putamen (145). In the same study, greater alcohol dependence severity was associated with lower striatal D2/3 availability. These findings suggest that dopaminergic abnormalities linked to AUD may already be detectable before the disorder is fully established, supporting their role as intermediate markers of risk rather than simply consequences of chronic alcohol exposure.

Conversely, some genetic variants associated with lower striatal D2/3 availability have been liked to increased risk. Carriers of the A1 allele of the Taq1A polymorphism (linked to the DRD2/ANKK1 locus), associated with reduced D2/3 receptor density, show higher risk for AUD; in an Indian male cohort, the A1 allele showed a trend toward association and a haplotype including A1 conferred an approximately 2.5-fold higher risk (146). In individuals with AUD, stimulant challenge paradigms consistently reveal blunted dopamine release in the ventral striatum (70, 147), a trait linked to higher craving, greater impulsivity and worse clinical outcomes (16). In unaffected relatives, dopamine release tends to resemble that of healthy controls (148). However, the expectation of receiving alcohol can potentiate ventral striatal dopamine release, with the strongest responses observed in those at higher genetic risk for AUD, consistent with an exaggerated anticipatory response to alcohol-related cues as a vulnerability phenotype (149).

Presynaptic variation also modulates dopaminergic imaging markers. DAT1 (SLC6A3) variable number tandem repeat (VNTR) genotype influences *in vivo* striatal DAT availability measured by ^123^I-*β*-CIT SPECT; individuals with the 9/10-repeat genotype show lower putaminal DAT availability than 10/10 homozygotes, while AUD status per se is not significantly associated with DAT availability (150). This suggests that DAT genetic variation can shape presynaptic dopaminergic tone and should be considered when interpreting presynaptic imaging biomarkers potentially relevant for vulnerability and clinical course in AUD (150).

Finally, cortical dopaminergic tone, strongly influenced by catechol-O-methyltransferase (COMT)—an enzyme that degrades dopamine in the prefrontal cortex—appears to impact impulsive choice. The COMT Val/Val genotype, associated with higher enzymatic activity and thus lower prefrontal dopamine, has been linked to a stronger preference for immediate over delayed rewards and to greater frontoparietal activity during intertemporal decision making, whereas Met carriers show the opposite pattern (151). These differences suggest that COMT modulates the efficiency of frontoparietal circuits involved in impulse control and may contribute to an impulsive intermediate phenotype relevant to clinical heterogeneity in AUD, potentially including an impulsive AUD subtype (151).

Taken together, these findings indicate that individual differences in dopaminergic receptor availability, presynaptic function, drug-induced release and cortical dopamine regulation—shaped in part by DRD2/ANKK1, DAT1 and COMT genotypes—contribute to both vulnerability and resilience to AUD. Integrating genetic and dopaminergic imaging markers may therefore help identify individuals at higher risk and refine biologically informed preventive and therapeutic strategies.

### 8.4 Opioid markers

In heavy drinkers, the magnitude of alcohol-induced endogenous opioid release in ventral striatum and OFC has been related to both the intensity of hedonic responses and drinking behavior (37). Larger alcohol-induced reductions in *μ*-opioid receptor binding potential (reflecting greater endogenous opioid release) have been associated with a stronger subjective “high” and with higher-risk drinking patterns, suggesting that heightened opioid hedonic sensitivity to alcohol may represent a risk phenotype rather than a mere state marker—that is, individuals who experience stronger initial reinforcement may be more likely to escalate their use (16, 37).

In addition, dopaminergic responses to alcohol appear to be modulated by genetic variation in the *μ*-opioid receptor gene (OPRM1). Carriers of the OPRM1 A118G (Asn40Asp/Asp40) variant have shown an enhanced alcohol-induced striatal dopamine response, consistent with the idea that interindividual differences in μ-opioid signaling can amplify reward-related responses to alcohol, although available studies remain limited and its role as a risk factor for AUD remains debated (152). Together, these findings support a model in which variability in μ-opioid function, shaped in part by OPRM1 genotype, contributes to heterogeneity in alcohol reward processing and may influence vulnerability and treatment response in AUD (152).

### 8.5 GABA markers and circuit-level vulnerability

In the GABAergic system, several genetic studies have identified variants in GABA_A_ receptor subunit genes associated with risk for AUD, with particularly strong evidence for the α2 subunit gene (GABRA2) (11, 153). Experimental and imaging data suggest that carriers of GABRA2 risk alleles report stronger stimulation and “high” following alcohol administration and show greater medial frontal activation to alcohol cues in fMRI, supporting the hypothesis that this variant enhances the reinforcing impact and salience of alcohol-related signals and thereby increases vulnerability to heavy drinking (153, 154).

A longitudinal fMRI study using a monetary incentive delay task in adolescents with high familial risk for AUD found that carriers of the minor GABRA2 rs279858 G allele exhibited exaggerated NAc activation to reward cues compared with non-carriers. This genetically mediated striatal hyperreactivity was associated with greater alcohol-related problems, and NAc activation statistically mediated the relationship between genotype and alcohol problems (11). These findings suggest that GABA_A_-related genetic variation may bias the reward circuitry toward heightened responsiveness to incentives, constituting an endophenotype that increases risk for AUD.

A recent multimodal study combining striatal D2/3 PET (^18^F-fallypride) with ACC GABA MRS further illustrates how converging alterations in inhibitory and dopaminergic systems may shape vulnerability. In healthy low-risk subjects, higher ACC GABA levels were associated with lower D2/3 receptor availability in the associative striatum, consistent with coordinated inhibitory–dopaminergic coupling. In individuals with AUD and in high-risk relatives, this negative correlation was not observed, suggesting a decoupling between cortical GABAergic tone and striatal dopaminergic signaling (155). Overall, these results are consistent with the hypothesis that, in AUD or familial vulnerability, disrupted inhibitory–dopaminergic coupling may reflect impaired cortical regulatory mechanisms over mesolimbic dopamine signaling relevant to addiction pathophysiology.

### 8.6 From prognostic markers to precision treatment targets

From a prognostic standpoint, several of the alterations described above carry information about relapse risk and clinical evolution. In early abstinence, greater cue-induced activation of medial prefrontal cortex, ACC and striatal regions in response to alcohol-related stimuli has been associated with a higher risk of relapse (13).

Resting-state connectivity involving executive-control and salience-related circuits has been related to craving intensity and treatment outcome in AUD (58). Additionally, voxel-wise connectivity density in heavy drinkers has been linked to individual differences in executive-control performance, a domain relevant to clinical outcome (55). Structurally, longitudinal MRI studies show whole-brain tissue volume gains with sustained abstinence and rapid reversal with resumed drinking. DBM further indicates that focal nodes of the fronto-ponto–cerebellar circuit, along with callosal and cortical regions, recover more in abstainers than in relapsers, suggesting that integrity of these pathways tracks structural brain recovery (6, 42). Finally, persistent frontal hypoperfusion and hypometabolism on ASL, SPECT and FDG-PET during early abstinence have been associated with poorer neuropsychological outcome and more frequent relapse, suggesting that multimodal structural, functional and metabolic markers may help identify patients at higher risk and guide the intensity of follow-up (156–158).

Genetic factors do not only influence the risk of developing AUD, but can also modulate the efficacy of therapeutic interventions, including pharmacological, behavioral and neuromodulatory approaches. Recent work has begun to link dopaminergic and GABAergic variants to differences in brain and clinical responses to treatments for AUD (159–161).

Building on this, a randomized, placebo-controlled pharmacogenetic fMRI trial tested the COMT inhibitor tolcapone in non-treatment-seeking individuals with AUD prospectively genotyped for COMT rs4680. Tolcapone significantly reduced alcohol consumption during treatment only in Val/Val homozygotes; it also decreased inferior frontal reactivity to alcohol cues, and lower inferior frontal activation on treatment was associated with less drinking (162). These findings suggest that enhancing cortical dopamine via COMT inhibition may improve control over intake in individuals with genetically mediated lower prefrontal dopaminergic tone, illustrating how dopaminergic genotype could inform personalized therapies in AUD (151, 162).

GABA_A_-related genetic variation may be relevant for individual differences in alcohol-related phenotypes that could influence treatment response. Experimental work indicates that GABRA2 risk alleles are associated with more intense subjective responses to administered alcohol (153). Although large clinical trials stratified by GABA genotype are still lacking, emerging evidence suggests that GABA_A_ subunit variants may influence treatment outcomes in AUD (159, 163), but whether they modulate responsiveness to sedative medications or neuromodulatory interventions targeting corticolimbic inhibitory networks remains to be tested.

Non-pharmacological interventions such as repetitive transcranial magnetic stimulation (rTMS) and deep brain stimulation (DBS) are being explored in AUD to modulate activity in regions involved in craving and control, such as the dlPFC or NAc (15, 164, 165). Although genotype-stratified trials have not yet been reported, interindividual variability in dopaminergic and GABAergic genes could influence the plasticity induced by these techniques. For instance, individuals with low prefrontal dopamine (COMT Val/Val) might benefit more from neuromodulation protocols that facilitate prefrontal activity, whereas those carrying variants associated with heightened mesolimbic reactivity (e.g., risk GABRA2 alleles) might require specifically tuned protocols to counteract an exaggerated reward response. While still speculative, this line of reasoning underscores the importance of incorporating genetic markers in future neuromodulation studies in AUD.

In summary, the integration of genetics and neuroimaging is beginning to clarify why some individuals respond better to specific therapies for AUD. Neurofunctional markers can be used to monitor treatment effects across genotypes, with the long-term goal of combining genomic characterization and imaging biomarkers to identify biologically distinct AUD subtypes and match them to the pharmacological, behavioral or neuromodulatory interventions with the highest probability of success in each case.

## 9 Methodological considerations, limitations and future directions

Most neuroimaging studies in alcohol use and AUD are still limited by relatively small samples, cross-sectional designs and heterogeneous clinical profiles. Many cohorts mix current and former drinkers, variable durations of abstinence, polysubstance use (especially nicotine and cannabis), psychotropic medications and comorbid psychiatric disorders, all of which can influence brain structure and function and contribute to between-study heterogeneity (68). Nutritional status, liver disease and sex-related factors are additional sources of variance that are rarely characterized in detail. These constraints reduce statistical power and may partly explain discrepancies between studies. Future work will benefit from larger, longitudinal and carefully phenotyped samples that distinguish between patterns of use (e.g., binge vs. continuous drinking), age at onset, sex and comorbidities, and that incorporate dimensional measures of craving, affective symptoms and executive dysfunction rather than relying solely on categorical diagnostic frameworks (1–3, 15).

An additional methodological issue is that structural brain loss may influence the interpretation of molecular PET findings in AUD. Because chronic alcohol use is associated with cortical thinning and regional volume loss—particularly in frontal cortex, hippocampus and cerebellum—partial volume effects may contribute to underestimation of tracer binding or metabolism in atrophic regions. Thus, apparent reductions in receptor availability or metabolic signal should not always be interpreted as purely molecular changes, especially when structural correction is not applied or is handled inconsistently across studies.

Kinetic modeling also presents specific challenges in AUD. Many molecular PET studies rely on reference-tissue approaches, yet identifying a truly unaffected reference region is difficult in a disorder characterized by widespread alcohol-related brain changes. This issue is particularly relevant for tracers quantified using approaches that rely on a reference region, including dopaminergic ligands using the cerebellum, ^11^C-ABP688 using cerebellar gray matter, ^11^C-carfentanil using occipital cortex, and more recently ^11^C-UCB-J using centrum semiovale, where even subtle alcohol-related abnormalities in the reference region could bias simplified binding estimates. Differences in the use of arterial input functions, metabolite correction, and partial volume correction further complicate comparisons across studies and may contribute to heterogeneity in reported effect sizes. These methodological issues should be considered when interpreting receptor PET findings in AUD and when comparing results across tracers, regions and stages of abstinence.

At the same time, emerging molecular PET approaches offer opportunities to refine mechanistic models and move toward clinically actionable biomarkers. PET tracers targeting neuroimmune processes (e.g., TSPO and potentially more specific next-generation glial markers) and synaptic density (SV2A) may help clarify to what extent alcohol-related brain changes reflect reversible synaptic downscaling, neuroimmune remodeling or irreversible neurodegeneration, and whether these components differ across clinical subtypes or stages of disease. Advanced connectomic approaches and graph-theoretical analyses can characterize how alcohol alters the topology of structural and functional networks beyond regional changes, potentially yielding network-level signatures of vulnerability and resilience. Machine-learning and multivariate methods applied to multimodal datasets—combining structural, functional, molecular and genetic information—may contribute to identifying candidate biotypes with distinct risk trajectories and treatment needs, and to generating predictive models of relapse or treatment response that can be prospectively tested (166).

Finally, the translation of neuroimaging markers into precision treatment must address issues of feasibility, cost, generalizability and ethical use. Biomarkers need to demonstrate incremental value over clinical assessment, robustness across scanners and sites, and stability over time. It is unlikely that a single imaging test will emerge; more plausibly, clinically useful tools will derive from relatively simple, standardized readouts—such as a limited set of network measures or regionally specific metabolic patterns—that can be combined with genetic and clinical variables in stratification algorithms. In this context, ongoing efforts to harmonize acquisition, preprocessing and reporting standards, together with prospective, hypothesis-driven validation of candidate markers in treatment trials, will be essential for integrating neuroimaging into routine management of AUD instead of confining it to the research realm.

## 10 Conclusion

Multimodal neuroimaging has delineated consistent patterns of alcohol-related brain involvement. Structural MRI and DTI implicate frontocerebellar, callosal and limbic pathways, with atrophy–partial recovery trajectories that depend substantially on abstinence. Perfusion imaging, FDG-PET and fMRI show acute uncoupling of flow, metabolism and neural activity evolving toward chronic hypoperfusion/hypometabolism in prefrontal, limbic and cerebellar networks in established AUD. Molecular imaging and MRS further support abnormalities in dopaminergic, opioid, GABAergic, glutamatergic and endocannabinoid systems within ventral striatum, prefrontal cortex, ACC, insula and cerebellum, broadly reflecting an imbalance between reward drive and inhibitory control, modulated by genetic variation and only partially reversible with abstinence.

Adolescent and emerging-adult binge drinking is associated with deviations from normative trajectories of cortical thinning, white-matter maturation and functional tuning of reward and control networks, even in the absence of formal AUD, and these changes have been linked to executive dysfunction and escalation of use. At the severe end of the spectrum, structural and perfusion imaging delineate overt damage in cerebellar and limbic circuits in alcohol-related neurological syndromes, providing essential information for diagnosis and management.

Across modalities, several signatures emerge as candidate biomarkers of vulnerability and prognosis: trait-like differences in dopaminergic and GABAergic circuits (including GABRA2, OPRM1, DRD2/ANKK1, DAT1 and COMT-related phenotypes), low subjective response to alcohol, cue-reactivity and resting-state profiles that predict relapse, and structural/metabolic indices of frontal and frontocerebellar integrity that track recovery. Integrating these measures with genetic and clinical data is beginning to define biologically informed subtypes of AUD and to identify patients more likely to benefit from specific pharmacological or neuromodulatory interventions. Progress will depend on larger, longitudinal better phenotyped cohorts, harmonized acquisition and analysis pipelines, and prospective validation of candidate markers in treatment studies. Emerging tools—neuroinflammation and synaptic-density PET tracers, advanced connectomics and machine-learning applied to multimodal datasets—offer a promising route toward more robust, network-level signatures that are feasible to implement, with the overarching aim of providing clinically actionable information for risk stratification, prognosis and precision treatment in AUD.
